# Background/CASPAR study

**DOI:** 10.1186/1470-7330-15-S1-O1

**Published:** 2015-10-02

**Authors:** Diana Tait

**Affiliations:** 1 The Royal Marsden NHS Foundation Trust, London, UK

## 

Accurate staging of cancer underpins the decision making for individual patients, the collection of outcomes data and the development of appropriate services. Unfortunately, this is not something that the wider oncology community has done well and there are many valid reasons why this part of cancer patient management has proved challenging. However, there are ways in which Clinical Radiologists can improve on staging reporting and contribute to better patient care. The CASPAR project was set up to explore the value of, and the processes necessary, for proforma reporting in common cancers.

Audits of histopathology reporting of colorectal cancer stage have shown an increase in minimum staging data from 31%-100% following the introduction of proforma reporting. As a consequence, minimum data-set reporting of prognostic histopathological data for resected cancers has become a global standard of care. The impact that this has on clinical outcomes is evident from subsequent studies which have shown that patients with staging reports where data set items are missing have poorer survival outcomes.

Imaging reporting, and the accurate identification of tumour stage, plays a critical role in the allocation of patients to appropriate pathways, particularly in those cancers where preoperative strategies are employed for identifiable groups of patients.

The CASPAR project sought to test the hypothesis that reporting practice and staging could improve through the implementation of proformas. The results will be discussed during this meeting but they do show an absolute improvement of 40% in hospitals that successfully made the implementation. Barriers to implementation are multifactorial but there are important issues with regard to technical capability and workforce availability.

National data collection and entry into research trials requires accurate staging and both are likely to be enhanced by the consistency, completeness and accuracy that structured proformas encourage.

